# Protective effects of melatonin in cisplatin-induced cardiac toxicity: possible role of BDNF-TNF-α signaling pathway

**DOI:** 10.1590/acb370208

**Published:** 2022-05-02

**Authors:** Xiaoqing Zhuo, Honglei Jiang

**Affiliations:** 1 MM. Department of Cardiology - Shandong Second Provincial General Hospital (Shandong ENT Hospital) - Jinan, China.

**Keywords:** Melatonin, Cisplatin, Glutathione, Brain-Derived Neurotrophic Factor, Rats

## Abstract

**Purpose:**

The present study explored the role of melatonin in cisplatin-induced cardiac injury along with the possible role of brain-derived neurotrophic factor (BDNF) in melatonin-mediated effects.

**Methods:**

Wistar rats were administered cisplatin (10 mg/kg), and cardiac injury was assessed by measuring the levels of cardiac troponin (cTnT) and lactate dehydrogenase (LDH-1).The extent of apoptosis was measured by measuring caspase-3 (pro-apoptotic) and Bcl-2 (anti-apoptotic) in hearts. The levels of BDNF, tumour necrosis factor α (TNF-α) and reduced glutathione were measured in heart. Melatonin (5 and 10 mg/kg) was administered for 15 days, and the role of BDNF was identified by co-administering BDNF inhibitor, ANA-12 (0.25 and 0.5 mg/kg).

**Results:**

Melatonin attenuated cTnT and LDH-1 levels along with reduction in caspase-3 and increase in Bcl-2. It also increased cisplatin-induced decrease in BDNF, increase in TNF-α and decrease in reduced glutathione levels. Moreover, ANA-12 abolished the cardioprotective effects, anti-inflammatory and antioxidant effects of melatonin suggesting the role of BDNF in melatonin-mediated effects in cisplatin-induced cardiac injury.

**Conclusions:**

Melatonin is useful in cisplatin-induced cardiac injury, which may be due to an increase in BDNF, decrease in inflammation and increase in antioxidant activities.

## Introduction

Chemotherapeutic agents are increasingly employed in patients due to the increase in the number of cancer patients. Amongst chemotherapeutic agents, cisplatin is very commonly used in number of malignancies such as including bladder, head and neck, lung, ovarian, and testicular cancers^1^. However, cisplatin is associated with a number of adverse effects including nephrotoxicity^2^, neurotoxicity^3^ and ototoxicity^4^. Cardiac injury is one of the important side effects of cisplatin^5^^,^ 6, and it may severely hamper the use of cisplatin in cancer patients. Therefore, there is a need to identify new targets or new therapeutic interventions to prevent or attenuate deleterious effects of cisplatin on the heart.

Melatonin is a neurohormone which is secreted by pineal gland and is reported to maintain circadian cycle in the body^7^. Apart from that, studies have explore the multifunctional role of melatonin in the brain including neuroprotective action in chemical-induced brain damage^8^, traumatic brain injury^9^, ischemic brain injury^10^ and neurodegeneration^11^. The protective functions of melatonin have also been identified in the heart^12^. It is reported to produce beneficial effects in heart failure^13^, ischemia-reperfusion induced myocardial injury^14^ and diabetic cardiomyopathy^15^. Based on these reports, it was hypothesized that melatonin may possibly produce protective actions in cisplatin-induced cardiac injury.

Brain-derived neurotrophic factor (BDNF) is a neurotrophic factor, and its role in producing neuroprotection has been well established^16^^,^ 17. Moreover, it has also been shown to confer cardioprotection in ischemia-reperfusion injury^18^^,^ 19. However, its role in cisplatin-induced cardiac injury has not been explored. Based on this, the present study was designed to explore the role of melatonin in cisplatin-induced cardiac injury along with the possible role of BDNF in melatonin-mediated protective effects in rats.

## Methods

### Animals and drugs

Male Wistar albino rats (200-250 g) were employed and kept in the animal house of an institute. Since there is hormonal variation in females that may alter the physiological and pathological effects, only male rats were employed in this study. The standard animal house conditions were met, and all experiments were performed as ethical norms of the Institutional Ethical Committee. The experimental protocols were approved by Shandong Second Provincial General Hospital (Shandong ENT Hospital) animal ethical committee, approval No. XYK20210112. The doses of melatonin^20^^,^ 21 and ANA-12^20^ were selected according to previously published studies.

### Cisplatin-induced cardiac injury model

A single dose of cisplatin (10 mg/kg *i.p.*) was given to rats to induce cardiac injury, and the extent of injury was assessed after five days on injection^21^.

### Assessment of cardiac injury parameters in plasma

Cardiac troponin (cTnT) and lactate dehydrogenase (LDH-1) are specific biomarkers of cardiac injury and widely employed in clinical and animal studies to assess the extent of cardiac injury^22^^,^ 23. The release of these markers in the plasma is an indication of cardiac injury, and their levels are in proportion to extent of cardiac injury. The levels of cTnT in the plasma were measured using a sandwich enzyme-linked immunosorbent assay (ELISA) kit (ab246529; Abcam Inc, Toronto, Canada), and the levels of LDH-1 in the plasma were measured by a colorimetric method using a commercially available kit (ab102526; Abcam Inc, Toronto, Canada).

### Preparation of heart homogenates

After five days of cisplatin injection, rats were sacrificed to isolate hearts. Rats were sacrificed by an overdose of 4.5% isoflurane (gaseous anaesthetic). The hearts were homogenised in a buffer solution (phosphate buffer solution–PBS), and the solution was centrifuged at 5,000 g for 15 minutes at 4°C to obtain a clear supernatant. The supernatant was employed to quantify the biochemical parameters in heart.

### Assessment of apoptosis in heart

Apoptosis constitutes an important type of cell death, and induction of apoptosis is well reported to participate in cardiac injury^24^. The extent of activation of apoptosis was assessed by measuring the activities of pro-apoptotic caspase-3 and anti-apoptotic, Bcl-2 in the heart homogenates. The increase in caspase-3 and the decrease in Bcl-2 activities indicate the induction of apoptosis. The caspase-3 activity was done using colorimetric assay kit (ab39401; Abcam Inc, Toronto, Canada), which was based on the formation of chromophore p-nitroaniline (p-NA) from DEVD-pNA in the presence of caspase-3 enzyme. The absorbance of p-NA was measured at 405 nm using a spectrophotometer. The quantification of Bcl-2 was done using commercially available ELISA kit (MBS2881713, MyBioSource, Inc. CA, United States of America).

### Assessment of BDNF, TNF-α and reduced glutathione levels in heart

The levels of BDNF (ab213899; Abcam Inc, Toronto, Canada) and tumour necrosis factor α (TNF-α) (ab46070; Abcam Inc, Toronto, Canada) were measured by commercially available sandwich based-ELISA kits, and procedure was adapted as the instructions of these kits. The levels of reduced glutathione levels were quantified by fluorometric assay kit (ab235670; Abcam Inc, Toronto, Canada). It was based on the formation of fluorescent product as a result of a reaction between reduced glutathione and a fluorophore. The intensity of fluorescence was measured at Excitation/Emission=535/587 nm, and the intensity was directly proportional to the amount of reduced glutathione in the sample.

### Experimental protocol

Six groups were employed, and each group comprised of eight animals. Thus, total 48 animals were employed.

Normal rats: They were kept for 15 days, and no intervention was given. On 15^th^ day, these rats were killed for isolation of heart and removal of blood for biochemical analysis;

Cisplatin-injected rats: On the 10^th^ day of protocol, a single injection of cisplatin (10 mg/kg) was given to rats to induce acute cardiac injury. Rats were sacrificed on 15^th^ day to isolate heart and blood for analysis;

Melatonin (5 mg/kg) in cisplatin-injected rats: Rats were treated with melatonin (5 mg/kg oral) for 15 days of experimental study. On the 10^th^ day, cisplatin (10 mg/kg) was given, and rest of protocol was same as in group 2;

Melatonin (10 mg/kg) in cisplatin-injected rats: Rats were treated with melatonin (10 mg/kg oral for 15 days of experimental study. On the 10^th^ day, cisplatin (10 mg/kg) was given, and the rest of protocol was same as in group 2.

ANA-12 (0.25 mg/kg) and melatonin (10 mg/kg) in cisplatin-injected rats: ANA-12 (0.25 mg/kg) was co-administered along with melatonin (10 mg/kg) for 15 days. The rest of procedure was same as in group 4;

ANA-12 (0.5 mg/kg) and melatonin (10 mg/kg) in cisplatin-injected rats: ANA-12 (0.5 mg/kg) was co-administered along with melatonin (10 mg/kg) for 15 days. The rest of procedure was same as in group 4.

### Statistical analysis

The GraphPad Prism 8 was employed for statistical analysis. The data were presented as mean ± standard deviation (SD). The data were statistically compared using one-way analysis of variance (ANOVA) followed by Tukey’s post hoc test. P<0.05 was considered as statistically significant.

## Results

### Effect of cisplatin and other pharmacological interventions on cardiac injury

**Figure 1:**
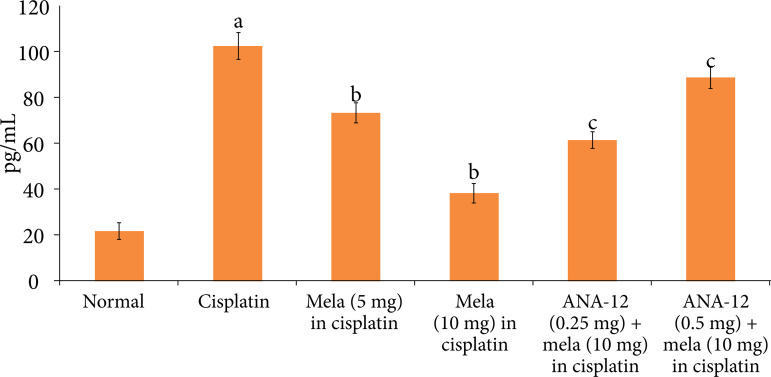
Effect of cisplatin and other pharmacological agents on the release of cTnT in the plasma. The values are in mean ± SD.

**Figure 2:**
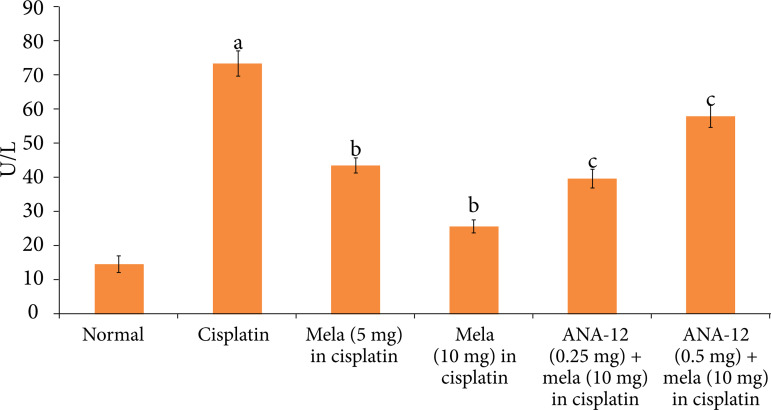
Effect of cisplatin and other pharmacological agents on the release of LDH-1 in the plasma. The values are in mean ± SD.

LDH-1: lactate dehydrogenase; Mela: melatonin; SD: standard deviation;a: *p*< 0.05 *vs*. normal; b: *p*< 0.05 *vs*. cisplatin; c: *p*< 0.05 *vs*. mela (10 mg) in cisplatin.

**Figure 3:**
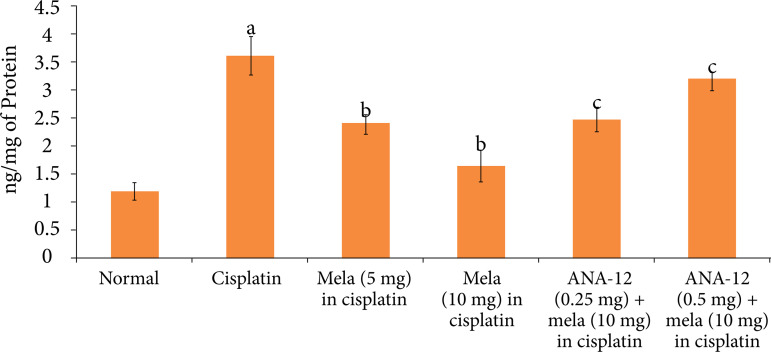
Effect of cisplatin and other pharmacological agents on the caspase-3 activity in the heart homogenates. The values are in mean ± SD.

**Figure 4:**
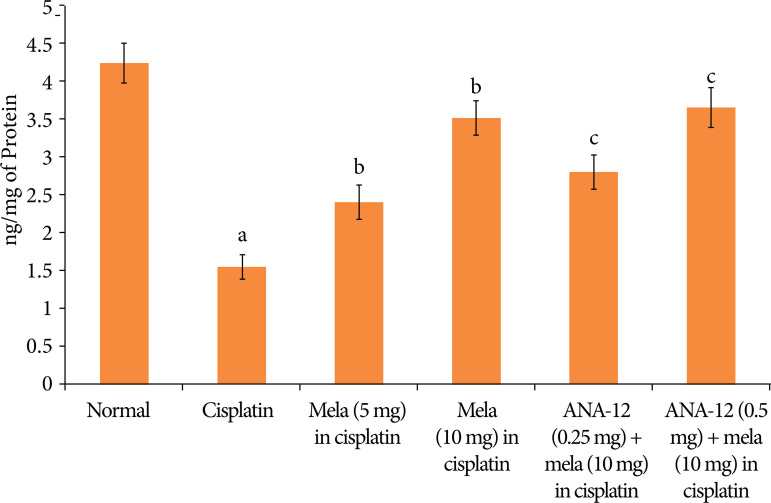
Effect of cisplatin and other pharmacological agents on the Bcl-2 activity in the heart homogenates. The values are in Mean ± SD.

A single dose of cisplatin led to significant myocardial injury, which was assessed on the fifth day of cisplatin administration (15^th^ day of experimental protocol). Indeed, there was a significant increase in the levels of cTnT (Fig. 1) and LDH-1 (Fig. 2) in the plasma following cisplatin administration in comparison to normal rats. Moreover, there was a significant increase in the caspase-3 activity (Fig. 3) and decrease in the Bcl-2 activity (Fig. 4) in the homogenates of cisplatin-injected rat hearts, suggesting the significant increase in apoptosis in these hearts.

cTnT: cardiac troponin; Mela: melatonin; SD: standard deviation;a: *p*< 0.05 *vs*. normal; b: *p*< 0.05 *vs*. cisplatin; c: *p*< 0.05 *vs*. mela (10 mg) in cisplatin.

Mela: melatonin; SD: standard deviation; a: *p*< 0.05 *vs*. normal;b: *p*< 0.05 *vs*. cisplatin; c: *p*< 0.05 *vs*. mela (10 mg) in cisplatin.

Mela: melatonin; SD: standard deviation; a: *p*< 0.05 *vs*. normal;b: *p*< 0.05 *vs*. cisplatin; c: *p*< 0.05 *vs*. mela (10 mg) in cisplatin.

Treatment with melatonin (5 and 10 mg/Kg oral) for 15 days, 10 days before cisplatin injection and five days after cisplatin injection significantly attenuated cisplatin-induced cardiac injury in a dose-dependent manner. There was a significant decline in the levels of cTnT (Fig. 1) and LDH-1 (Fig. 2), suggesting the cardioprotection in melatonin-treated rats. Moreover, there was a significant decrease in the caspase-3 activity (Fig. 3) and increase in Bcl-2 activity (Fig. 4) in melatonin-treated rats, suggesting the significant reduction in apoptosis. However, co-administration of ANA-12 (0.25 and 0.50 mg/kg), BDNF blocker, significantly attenuated the cardioprotective and anti-apoptotic effects of melatonin (10 mg/Kg) in a significant manner, and there was a rise in the cTnT levels, LDH-1 levels, increase in caspase-3 activity and decrease in Bcl-2 activity in ANA-12 treated rats in a dose-dependent manner.

**Figure 5:**
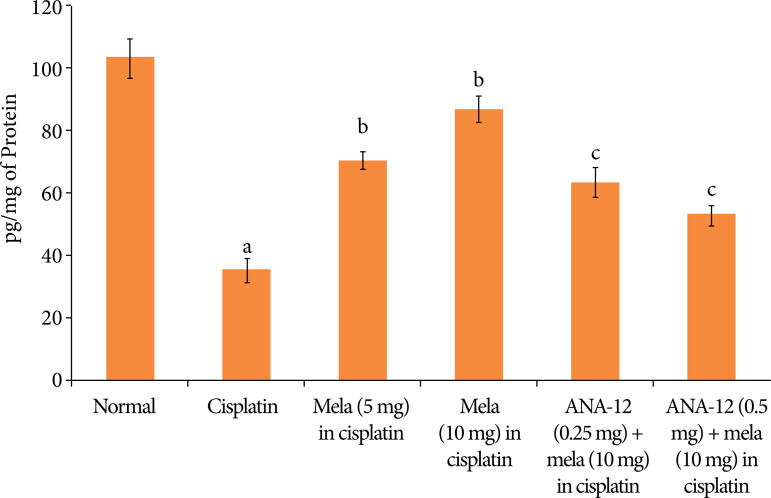
Effect of cisplatin and other pharmacological agents on the levels of BDNF in the heart homogenates. The values are in means ± SD.

BDNF: brain-derived neurotrophic factor; Mela: melatonin; SD: standard deviation;a: *p*< 0.05 *vs*. normal; b: *p*< 0.05 *vs*. cisplatin; c: *p*< 0.05 *vs*. mela (10 mg) in cisplatin.

**Figure 6:**
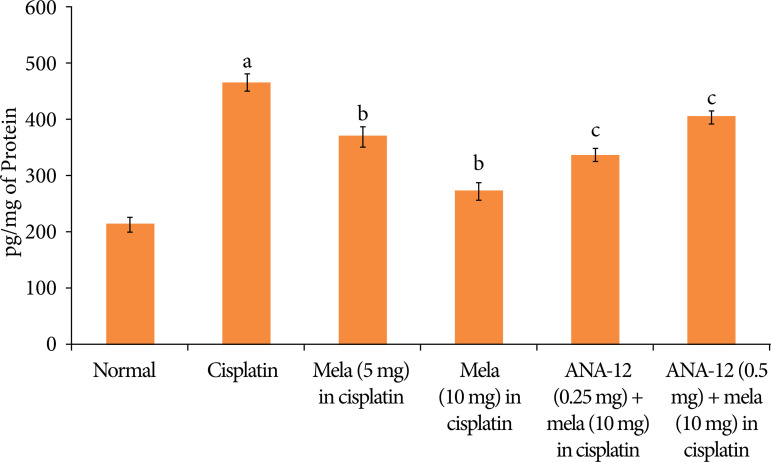
Effect of cisplatin and other pharmacological agents on the TNF-α levels in the heart homogenates. The values are in mean ± SD.

**Figure 7:**
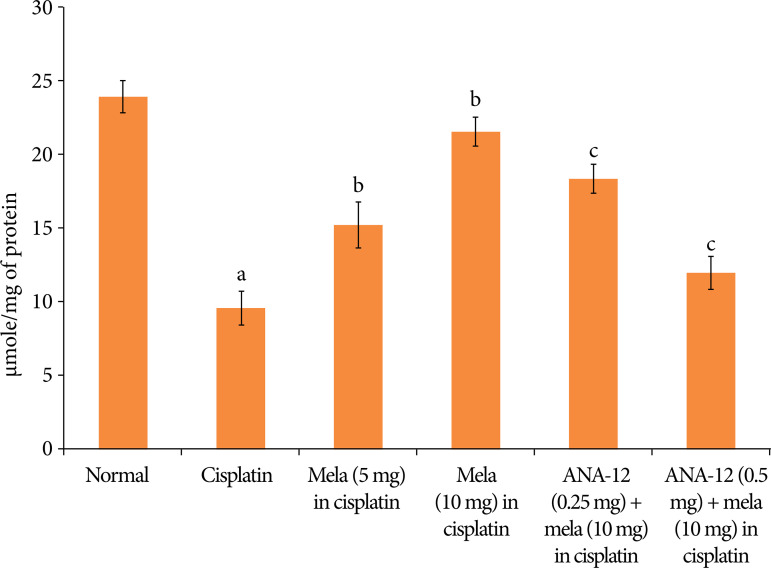
Effect of cisplatin and other pharmacological agents on the reduced glutathione levels in the heart homogenates. The values are in mean ± SD.

In cisplatin-injected rats, there was a significant decrease in the BDNF levels (Fig. 5), increase in the TNF-α levels (Fig. 6) and decrease in the reduced glutathione levels (Fig. 7). Treatment with melatonin (5 and 10 mg/kg) for 15 days significantly attenuated cisplatin-induced deleterious changes in the heart homogenates, and there was a significant restoration of BDNF levels, decrease in TNF-α and increase in the reduced glutathione levels. However, co-administration of ANA-12 (0.25 and 0.5 mg/kg) abrogated the restorative effects of melatonin on the biochemical parameters in cisplatin-injected rats in a significant manner.

TNF-α: tumour necrosis factor α; Mela: melatonin; SD: standard deviation;a: *p*< 0.05 *vs*. normal; b: *p*< 0.05 *vs*. cisplatin; c: *p*< 0.05 *vs*. mela (10 mg) in cisplatin.

Mela: melatonin; SD: standard deviation; a: *p*< 0.05 *vs*. normal;b: *p*< 0.05 *vs*. cisplatin; c: *p*< 0.05 *vs*. mela (10 mg) in cisplatin.

## Discussion

In the present study, a single dose injection of cisplatin led to significant cardiac injury as assessed by a significant increase in the cardiac injury biomarkers, i.e., cTnT and LDH-1, in the plasma of cisplatin-injected rats. Moreover, cisplatin also led to increase in apoptosis in rat hearts as assessed by increase in pro-apoptotic, caspase-3 activity and decrease in anti-apoptotic, Bcl-2 activity. It suggests that cisplatin may trigger apoptotic cell injury in the rat hearts to induce cardiac injury. Cisplatin is an antineoplastic drug and widely used in the treatment of different types of tumors^25^^,^ 26. However, chemotherapy with cisplatin leads to various side effects including increase in cardiac injury^27^^,^ 28. There are earlier studies showing that cisplatin leads to cardiac injury including increase in apoptosis in rats^21^.

In this investigation, treatment with melatonin for 15 days produced significant protection against cisplatin-induced cardiac injury. Indeed, in melatonin-treated rats, there was a significant decrease in cardiac injury biomarkers along with decrease in the apoptosis markers. These results suggest that melatonin has the cardioprotective potential against cisplatin-induced cardiac injury. Melatonin is a neurohormone and it is important in maintaining the circadian cycle^29^. In the cardiovascular system, melatonin has been shown to produce cardioprotective effects in cardiac heart failure^13^, ischemia-reperfusion injury^30^, sepsis-induced heart injury^31^ and calcium overload heart injury^32^. However, to best of our knowledge, it is the first study depicting the cardioprotective effects of melatonin in cisplatin-induced cardiac injury model in rats.

In the study, there was a significant decrease in the BDNF levels in the hearts of cisplatin-injected rats. BDNF is an important neurotrophic factor and, apart from its brain protective effects^33^, it has been shown to confer protection to heart in different disease models^18^^,^ 34. The decrease in BDNF levels in cisplatin-injected rats suggests that a decrease in the expression of BDNF may possibly contribute in inducing cisplatin-induced cardiac injury. However, treatment with melatonin significantly restored cisplatin-induced decrease in the expression of BDNF in the rat hearts. It possibly suggests that melatonin may increase the expression of BDNF to produce protection against cisplatin-induced cardiac injury. To further explore the possible role of BDNF in melatonin-mediated cardioprotection, ANA-12, a pharmacological antagonist of BDNF, was co-administered along with melatonin in cisplatin-injected rats. Co-administration of ANA-12 was shown to significantly abolish the cardioprotective effects and anti-apoptotic effects of melatonin in cisplatin-injected rats. It further suggests that melatonin-mediated increase in the BDNF levels may possibly contribute to cardioprotection in cisplatin-induced cardiac injury in rats. To best of our knowledge, it is the first study showing that melatonin may protect the heart from cisplatin-induced cardiac injury through an increase in BDNF expression.

Furthermore, in this study, cisplatin-induced cardiac injury was associated with an increase in the expression of inflammatory marker, i.e., TNF-α, and decrease in antioxidant activities, i.e., in reduced glutathione levels. An increase in inflammation and an decrease in antioxidant activities are crucial in inducing cardiac injury of diverse etiology including cisplatin-induced cardiac injury^35^. However, treatment with melatonin prevented rise in inflammation and decline in antioxidant activities in the rat hearts in cisplatin model. Previous studies have shown that melatonin has the potential to prevent inflammation^36^ and increase antioxidant activities in hearts^37^. Accordingly, it is possible to suggest that melatonin-mediated decrease in inflammation and increase in antioxidant activities may contribute in decreasing cisplatin-induced cardiac injury. Moreover, co-administration of ANA-12 abolished melatonin-induced decrease in inflammation and antioxidant activities, suggesting that melatonin-mediated anti-inflammatory and antioxidant effects may be possibly due to increase in BDNF levels. This contention is supported by the previous studies showing that BDNF decreases inflammation and increases antioxidant activities^38^^,^ 39. Accordingly, it may be proposed that melatonin may increase the expression of BDNF, which may contribute in decreasing inflammation and increasing antioxidant activities, and these actions may be important in conferring protection against cisplatin-induced cardiac injury in rats.

## Conclusion

Melatonin may be useful in attenuating cisplatin-induced cardiac injury, and these protective actions may be due to increase in the expression of BDNF, which may contribute in decreasing inflammation and increasing antioxidant activities.

## References

[1] Dasari S, Tchounwou PB (2014). Cisplatin in cancer therapy: molecular mechanisms of action. Eur J Pharmacol.

[2] Pabla N, Dong Z (2008). Cisplatin nephrotoxicity: mechanisms and renoprotective strategies. Kidney Int.

[3] Harmers FP, Gispen WH, Neijt JP (1991). Neurotoxic side-effects of cisplatin. Eur J Cancer.

[4] Santabarbara G, Maione P, Rossi A, Gridelli C (2016). Pharmacotherapeutic options for treating adverse effects of Cisplatin chemotherapy. Expert Opin Pharmacother.

[5] Gunturk EE, Yucel B, Gunturk I, Yazici C, Yay A, Kose K (2019). The effects of N-acetylcysteine on cisplatin induced cardiotoxicity. Bratisl Lek Listy.

[6] Stojic IM, Jakovljevic VL, Zivkovic VI, Srejovic IM, Nikolic TR, Jeremic JN, Jeremic NS, Djuric DM, Radonjic KG, Labudovic-Borovic M, Bugarcic ZD, Bogojeski J, Novokmet SS (2018). The perfusion of cisplatin and cisplatin structural analogues through the isolated rat heart: the effects on coronary flow and cardiodynamic parameters. Gen Physiol Biophys.

[7] Satyanarayanan SK, Su H, Lin Y-W, Su K-P (2018). Circadian rhythm and melatonin in the treatment of depression. Curr Pharm Des.

[8] Edres HA, Taha NM, Lebda MA, Elfeky MS (2021). The potential neuroprotective effect of allicin and melatonin in acrylamide-induced brain damage in rats. Environ Sci Pollut Res Int.

[9] Rehman SU, Ikram M, Ullah N, Alam SI, Park HY, Badshah H, Choe K, Kim MO (2019). Neurological enhancement effects of melatonin against brain injury-induced oxidative stress, neuroinflammation, and neurodegeneration via AMPK/CREB signaling. Cells.

[10] Ramos E, Patiño P, Reiter RJ, Gil-Martín E, Marco-Contelles J, Parada E, de Los, Romero A, Egea J (2017). Ischemic brain injury: new insights on the protective role of melatonin. Free Radic Biol Med.

[11] Hossain MF, Uddin MS, Uddin GMS, Sumsuzzman DM, Islam MS, Barreto GE, Mathew B, Ashraf GM (2019). Melatonin in Alzheimer’s disease: a latent endogenous regulator of neurogenesis to mitigate Alzheimer’s neuropathology. Mol Neurobiol.

[12] Sun H, Gusdon AM, Qu S (2016). Effects of melatonin on cardiovascular diseases: progress in the past year. Curr Opin Lipidol.

[13] Nduhirabandi F, Maarman GJ (2018). Melatonin in heart failure: a promising therapeutic strategy?. Molecules.

[14] Lan H, Su Y, Liu Y, Deng C, Wang J, Chen T, Jules KED, Masau JF, Li H, Wei X (2019). Melatonin protects circulatory death heart from ischemia/reperfusion injury via the JAK2/STAT3 signalling pathway. Life Sci.

[15] Song Y-J, Zhong C-B, Wu W (2020). Cardioprotective effects of melatonin: Focusing on its roles against diabetic cardiomyopathy. Biomed Pharmacother.

[16] Colucci-D’Amato L, Speranza L, Volpicelli F (2020). Neurotrophic factor BDNF, physiological functions and therapeutic potential in depression, neurodegeneration and brain cancer. Int J Mol Sci.

[17] Jin H, Zhu Y, Li Y, Ding X, Ma W, Han X, Wang B (2019). BDNF-mediated mitophagy alleviates high-glucose-induced brain microvascular endothelial cell injury. Apoptosis.

[18] Wang Z, Wang SP, Shao Q, Li PF, Sun Y, Luo LZ, Yan XQ, Fan ZY, Hu J, Zhao J, Hang PZ, Du ZM (2019). Brain-derived neurotrophic factor mimetic, 7,8-dihydroxyflavone, protects against myocardial ischemia by rebalancing optic atrophy 1 processing. Free Radic Biol Med.

[19] Wang G, Li Y, Lei C, Lei X, Zhu X, Yang L, Zhang R (2021). Quercetin exerts antidepressant and cardioprotective effects in estrogen receptor α-deficient female mice via BDNF-AKT/ERK1/2 signaling. J Steroid Biochem Mol Biol.

[20] Jiang R, Wei H (2021). Beneficial effects of octreotide in alcohol-induced neuropathic pain. Role of H 2S, BDNF, TNF-α and Nrf2. Acta Cir Bras.

[21] Saleh DO, Mansour DF, Mostafa RE (2020). Rosuvastatin and simvastatin attenuate cisplatin-induced cardiotoxicity via disruption of endoplasmic reticulum stress-mediated apoptotic death in rats: targeting ER-Chaperone GRP78 and Calpain-1 pathways. Toxicol Rep.

[22] Garg P, Morris P, Fazlanie AL, Vijayan S, Dancso B, Dastidar AG, Plein S, Mueller C, Haaf P (2017). Cardiac biomarkers of acute coronary syndrome: from history to high-sensitivity cardiac troponin. Intern Emerg Med.

[23] Navarro-Sobrino M, Lorita J, Soley M, Ramírez I (2010). Catecholamine-induced heart injury in mice: differential effects of isoproterenol and phenylephrine. Histol Histopathol.

[24] Zhang W-X, He B-M, Wu Y, Qiao J-F, Peng Z-Y (2019). Melatonin protects against sepsis-induced cardiac dysfunction by regulating apoptosis and autophagy via activation of SIRT1 in mice. Life Sci.

[25] Mohiuddin MD, Kasahara K (2021). Cisplatin and pemetrexed have distinctive growth-inhibitory effects in monotherapy and combination therapy on KRAS-dependent A549 lung cancer cells. Cancer Genomics Proteomics.

[26] Meirovitz A, Bergerson S, Hirshoren N, Weinberger JM, Bersudski E, Daniel S, Sheva K, Perez CA (2021). Modified bi-weekly cetuximab-cisplatin and 5-FU/leucovorin based regimen for effective treatment of recurrent/metastatic head and neck squamous cell carcinoma to reduce chemotherapy exposure of patients. Cancer Rep (Hoboken).

[27] Kim C-W, Choi K-C (2021). Effects of anticancer drugs on the cardiac mitochondrial toxicity and their underlying mechanisms for novel cardiac protective strategies. Life Sci.

[28] Lin Z, Bao Y, Hong B, Wang Y, Zhang X, Wu Y (2021). Salvianolic acid B attenuated cisplatin-induced cardiac injury and oxidative stress via modulating Nrf2 signal pathway. J Toxicol Sci.

[29] Claustrat B, Leston J (2015). Melatonin: physiological effects in humans. Neurochirurgie.

[30] Ma Z, Xin Z, Di W, Yan X, Li X, Reiter RJ, Yang Y (2017). Melatonin and mitochondrial function during ischemia/reperfusion injury. Cell Mol Life Sci.

[31] Rahim I, Sayed RK, Fernández-Ortiz M, Aranda-Martínez P, Guerra-Librero A, Fernández-Martínez J, Rusanova I, Escames G, Djerdjouri B, Acuña-Castroviejo D (2021). Melatonin alleviates sepsis-induced heart injury through activating the Nrf2 pathway and inhibiting the NLRP3 inflammasome. Naunyn Schmiedebergs Arch Pharmacol.

[32] Kong L, Wei M, Sun N, Zhu J, Su X (2017). Role of melatonin in calcium overload-induced heart injury. Zhong Nan Da Xue Xue Bao Yi Xue Ban.

[33] Marosi K, Mattson MP (2014). BDNF mediates adaptive brain and body responses to energetic challenges. Trends Endocrinol Metab.

[34] Hang P, Sun C, Guo J, Zhao J, Du Z (2016). BDNF-mediates Down-regulation of microRNA-195 inhibits ischemic cardiac apoptosis in rats. Int J Biol Sci.

[35] Xing JJ, Hou JG, Liu Y, Zhang RB, Jiang S, Ren S, Wang YP, Shen Q, Li W, Li XD, Wang Z (2019). Supplementation of saponins from leaves of panax quinquefolius mitigates cisplatin-evoked cardiotoxicity via inhibiting oxidative stress-associated inflammation and apoptosis in mice. Antioxidants (Basel).

[36] Huang CC, Chiou CH, Liu SC, Hu SL, Su CM, Tsai CH, Tang CH (2019). Melatonin attenuates TNF-α and IL-1β expression in synovial fibroblasts and diminishes cartilage degradation: Implications for the treatment of rheumatoid arthritis. J Pineal Res.

[37] Ishihara R, Barros MP, Silva CM, Borges L, Hatanaka E, Lambertucci RH (2021). Melatonin improves the antioxidant capacity in cardiac tissue of Wistar rats after exhaustive exercise. Free Radic Res.

[38] Han R, Liu Z, Sun N, Liu S, Li L, Shen Y, Xiu J, Xu Q (2019). BDNF Alleviates neuroinflammation in the hippocampus of type 1 diabetic mice via blocking the aberrant HMGB1/RAGE/NF-κB pathway. Aging Dis.

[39] Scotton E, Colombo R, Reis JC, Possebon GMP, Hizo GH, Valiati FE, Géa LP, Bristot G, Salvador M, Silva TM, Guerra AE, Lopes TF, Rosa AR, Kunz M (2020). BDNF prevents central oxidative damage in a chronic unpredictable mild stress model: the possible role of PRDX-1 in anhedonic behavior. Behav Brain Res.

